# 13 Identifying Risk Factors for Falls in Burn Patients: A Retrospective Analysis

**DOI:** 10.1093/jbcr/iraf019.013

**Published:** 2025-04-01

**Authors:** Tiffany Hockenberry, Isabella Hernandez, Meghana Tripuraneni, Suzanne Osborn, Stacey Richerbach, Natalie Kesler, Claudia Islas, Karen Richey, Kevin Foster

**Affiliations:** Diane & Bruce Halle Arizona Burn Center; Diane & Bruce Halle Arizona Burn Center; Diane & Bruce Halle Arizona Burn Center; Diane & Bruce Halle Arizona Burn Center; Diane & Bruce Halle Arizona Burn Center; Diane & Bruce Halle Arizona Burn Center; Diane & Bruce Halle Arizona Burn Center; Diane & Bruce Halle Arizona Burn Center; Diane & Bruce Halle Arizona Burn Center

## Abstract

**Introduction:**

It is essential healthcare facilities provide a safe environment for patients. Hospital falls can lengthen a patient’s recovery, increase hospital costs, and negatively affect a patient’s sense of security and confidence. Reducing falls may indicate better patient care practices, effective risk management, and ultimately a commitment to patient safety. Patients admitted for burn injuries tend to have higher scores on fall risk assessment tools. This complicates identification of patients who are truly at risk for falls within the burn population. The purpose of this study was to investigate potential fall risk factors unique to burn patients.

**Methods:**

This was a retrospective study of patients admitted to a burn center who sustained falls between 2017 and 2023. Data included demographics, burn characteristics, co-morbidities, medications, dressings, hemoglobin level, bed alarm use, staffing, and witnessed versus unwitnessed falls.

**Results:**

A total of 111 patients fell within the timeframe. Mean age was 48.9 years, 69% were male with an average BMI of 27.1. The population consisted of 78% Non-Hispanic Caucasian and Hispanic Caucasian. Most patients were admitted the day of their burn injury with 85% having a < 20% TBSA burn. The etiology was flash/flame in 42% and contact in 37%. Comorbidities included substance use disorders in 38%, and psychiatric disorders in 29%. Eight percent of patients fell multiple times. Patient falls were predominantly unwitnessed (66%) in their rooms (68%) or their bathroom (12%). Twenty-three % falls resulted in injury. In 69% of patients, a bed alarm was indicated, however of those the bed alarm was utilized in only 29%. Assistive devices were assigned to 39% of the patients who fell. These primarily front wheel walkers (61%). Eighty-nine percent had two or more invasive lines or tubes at time of the fall. Patient’s mean hemoglobin on day of fall was 10.48 indicating anemia was not a predisposing factor. Three-quarters (75%) of patients had a mind-altering medication given within a four-hour timeframe, and 60% of those received a narcotic or a benzodiazepine or both. The shift staffing assignments had an average variance of 3 hours per patient day, equating to a 16% reduction in budgeted staffing.

**Conclusions:**

The main risk factors identified in this assessment that may have attributed to the falls were lack of active bed alarms, administration of mind-altering medications within a four-hour timeframe, and understaffing. Additionally, the majority of patients that fell had a comorbidity of a psychiatric diagnosis or substance use disorder of some sort. Prior studies within the institution had demonstrated difficulty in caring for patients with substance use disorder.

**Applicability of Research to Practice:**

Creating a safe environment in acute care settings must remain a top priority in healthcare, including identifying risk factors that could lead to further harm, such as patient falls.

**Funding for the Study:**

N/A

